# Artificial Intelligence: A Snapshot of Its Application in Chronic Inflammatory and Autoimmune Skin Diseases

**DOI:** 10.3390/life14040516

**Published:** 2024-04-16

**Authors:** Federica Li Pomi, Vincenzo Papa, Francesco Borgia, Mario Vaccaro, Giovanni Pioggia, Sebastiano Gangemi

**Affiliations:** 1 Department of Precision Medicine in Medical, Surgical and Critical Care (Me.Pre.C.C.), University of Palermo, 90127 Palermo, Italy; federicalipomi@hotmail.it; 2 Department of Clinical and Experimental Medicine, School and Operative Unit of Allergy and Clinical Immunology, University of Messina, 98125 Messina, Italy; papavi.994@gmail.com (V.P.); sebastiano.gangemi@unime.it (S.G.); 3 Department of Clinical and Experimental Medicine, Section of Dermatology, University of Messina, 98125 Messina, Italy; mario.vaccaro@unime.it; 4 Institute for Biomedical Research and Innovation (IRIB), National Research Council of Italy (CNR), 98164 Messina, Italy; giovanni.pioggia@cnr.it

**Keywords:** artificial intelligence, machine learning, skin, autoimmune disease, inflammation, atopic dermatitis, psoriasis, vitiligo, alopecia areata, hidradenitis suppurativa

## Abstract

Immuno-correlated dermatological pathologies refer to skin disorders that are closely associated with immune system dysfunction or abnormal immune responses. Advancements in the field of artificial intelligence (AI) have shown promise in enhancing the diagnosis, management, and assessment of immuno-correlated dermatological pathologies. This intersection of dermatology and immunology plays a pivotal role in comprehending and addressing complex skin disorders with immune system involvement. The paper explores the knowledge known so far and the evolution and achievements of AI in diagnosis; discusses segmentation and the classification of medical images; and reviews existing challenges, in immunological-related skin diseases. From our review, the role of AI has emerged, especially in the analysis of images for both diagnostic and severity assessment purposes. Furthermore, the possibility of predicting patients’ response to therapies is emerging, in order to create tailored therapies.

## 1. Introduction

Immuno-correlated dermatological pathologies refer to skin disorders that are closely associated with immune system dysfunction or abnormal immune responses. These conditions involve an intricate interplay between the immune system and the skin, leading to various dermatological manifestations. Immuno-correlated dermatological pathologies encompass a broad spectrum of disorders, including autoimmune skin diseases, inflammatory skin conditions, and those associated with altered immune responses [1]. Examples of immuno-correlated dermatological pathologies include autoimmune blistering disorders such as pemphigus and bullous pemphigoid, inflammatory conditions like psoriasis and atopic dermatitis (AD), and connective tissue diseases such as lupus erythematosus. In these disorders, the immune system mistakenly targets components of the skin, resulting in inflammation, tissue damage, and characteristic skin lesions. Understanding the immune mechanisms underlying these dermatological conditions is crucial for developing effective diagnostic and therapeutic strategies. Moreover, advancements in the field of artificial intelligence (AI) have shown promise in enhancing the diagnosis, management, and assessment of immuno-correlated dermatological pathologies [2]. This intersection of dermatology and immunology plays a pivotal role in comprehending and addressing complex skin disorders with immune system involvement. Traditional diagnoses of dermatological diseases heavily rely on visual inspection and subjective evaluations, lacking precise, objective, and quantitative criteria. Dermatologists, despite their expertise, are not immune to misdiagnosis. In remote areas with limited access to dermatologists, non-specialists often handle dermatological diagnoses without extensive knowledge or training in the field. Even with dermatology textbooks as references, accurate diagnoses remain challenging. The scarcity of dermatologists and uneven healthcare resource distribution further complicate accurate diagnoses in these regions. AI technology based on image recognition has emerged as a promising approach for diagnosing skin diseases, addressing challenges in areas with limited healthcare resources. AI algorithms, trained on extensive datasets of skin images, excel in learning patterns associated with various skin conditions. This enables them to provide accurate diagnoses, particularly in the early stages of diseases [3]. Through meticulous design and debugging, AI algorithms may avoid biases inherent in human diagnoses, offering more objective results. Commonly used AI algorithms include machine learning (ML) and deep learning (DL), with DL showing superior performance in handling large datasets and complex features [4]. ML methods remain valuable in situations with limited data. These approaches find application in computer-aided diagnosis (CAD) systems, delivering precise classifications for dermatologists and aiding non-dermatologists in minimizing errors due to limited expertise [2]. The paper explores the evolution and achievements of ML and DL methods in diagnosis; discusses segmentation and the classification of medical images; and reviews existing challenges in immunological-related skin diseases. By comparing these methods and summarizing their limitations, the paper proposes future directions for development.

## 2. Results

### 2.1. Atopic Dermatitis

#### 2.1.1. AI Etiopathogenetic Application

Beyond the extensive interest in the field of allergopathies and immunological disorders, concerning AD and the already emphasized importance of data-driven methods applied to it, the first applicative evidence of AI using artificial neural networks (ANNs) dates to the last decade of the past century [5,6,7,8,9]. Regarding etiopathogenesis, an important contribution to the understanding of AD was recently offered by explainable AI, by which the dysregulation of the inhibitor of nuclear factor kappa B kinase subunit beta- nuclear factor kappa-light-chain-enhancer of activated B cells (Ikkb-NF-kB) axis in the paired related homeobox-1 (Prx1)+ fibroblastic subpopulation promoting skin inflammation by the overexpression of eotaxin-1 could be identified as a new unknown etiologic factor [10]. 

#### 2.1.2. Predictive, Diagnostic, and Classification Performances

For diagnostic purposes, only within the past decade has the interest in the clinical application potential of AI undergone a consistent revival, even more so with the use of data augmentation in DL-based approaches, increasing the accuracy of such models [11]. In 2015, Ghosh et al., through the use of AD “signature” genes (89 AD Gene Expression Signature, “89ADGES”) and the use of a support vector machine (SVM) for data analysis, built a final model with an AD predictive accuracy of 98% [12]. An interesting and recent extension of this topic comes from the use for classification purposes of ML models to identify common and distinct gene expression profiles between lesional areas and, especially, unique gene signatures between non-lesional areas of AD and those of other inflammatory skin diseases [13,14]. It is also intriguing to note how AI is increasingly impacting precision medicine for such disease in recent years, using unsupervised ML approaches designed to distinguish AD patients into various clusters, based on a different expression profile of multiple cytokines and chemokines, thus paving the way for the endotypic classification of AD and other allergic diseases [15]. Further impetus for the development of molecular diagnostics in AD is provided by the exploration of how deep-representation-learning techniques perform in analyzing large transcriptomic datasets for the prediction of phenotypes and, thus, clinical outcomes [16]. Likewise, unsupervised clustering approaches using hypothesis-independent statistical techniques for the identification of AD clinical phenotypes have also been attempted [17]. The latest advances in this topic involved the development of an ML classifier which, by employing it as input data on intestinal epithelial transcriptome and intestinal microbiome, was able to accurately and automatically classify AD, as well as identify its potential new biomarkers [18]. In addition, the attempt at identifying clinically relevant skin chemical biomarkers is strengthened by the integration of advanced ML methods to confocal Raman micro-spectroscopy, aiming to discriminate between AD subjects and healthy controls [19]. The utility of employing AI in the diagnosis of AD has continued to be increasingly investigated over the past 3 years [20,21,22,23]. We start from the promising results obtained by employing multiphoton tomography (MPT) imaging to train convolutional neural networks (CNNs) capable of recognizing living cells, and, through that, arrive at a rapid and operator-independent—in a single word, automatic—diagnosis of such a chronic skin condition [24]. Concurrently, Wu’s group, proposing a DL-based AI dermatology diagnosis assistant (AIDDA), obtained the same promising results with an AD diagnostic accuracy of 92.57%, with a specificity of 94.41% and sensitivity of 94.56% [25]. As if that were not sufficient, AI by means of CNNs has also approached high-frequency ultrasound skin imaging both through the proposal of a deep-transfer-learning-based algorithm able to classify with generally good accuracy various dermatoses, including AD, otherwise difficult to distinguish ultrasonographically, and by proposing DL-based models for the automated segmentation of skin layers [26,27,28]. Even more interestingly and recently, ML techniques have been employed to build predictive models of AD onset in childhood by analyzing large datasets including information related to prenatal exposure to environmental pollutants. The best predictive performance was recorded by the random forest (RF) model [29]. The year 2023, by itself, saw a flurry of work regarding the application of AI for improving AD expertise, starting with the proposal of new and efficient AD predictive models, such as bSRWPSO-FKNN by combining swarm intelligence algorithms (binary enhanced particle swarm optimization; bSRWPSO) with well-known supervised ML techniques (fuzzy K-nearest neighbor; FKNN) [30]. Following this, the introduction of DL models for the accurate classification of skin conditions, including AD, through the automatic extraction of lesions and segmented images of skin areas, enabled the creation of an image dataset useful for the performance enhancement in CAD of multiple skin conditions [31]. In this context, interestingly, the application of ML methods (with the optimal model represented by Extreme Gradient Boosting; XGB) is increasingly gaining ground for achieving an accurate diagnosis of AD by using biomarkers such as pyroptosis-related genes (PRGs) [32]. 

#### 2.1.3. A New Concept of AD Severity Scoring

In the wake of personalized dermatology and precision medicine, the most recent advancements have resulted both through ML-gradient boosting models for the identification of major AD-severity-associated factors and in the design of a Bayesian-ML-based probabilistic predictive model of the daily evolution of AD severity scores in the individual patient, thus optimizing the type and timing of treatment and, thereby, disease control [33,34,35]. The latest frontier of AI applied to Raman imaging for such a disorder has involved the use of DL analysis for the noninvasive quantification of the inflammatory response [36]. In the framework of better-defining the skin barrier function, a recent observational study employed an SVM model to classify eczema from data indicating the amount of three major natural skin-moisturizing components, thus enabling the formulation of a new quantitative index, the Eczema Biochemical Index (EBI), useful for staging disease severity [37]. Concerning the latter, very promising is the attempt to arrive at an automatic definition of AD severity by using CNNs being trained with brightness-adjusted clinical images to achieve a scoring accuracy of erythema, papulation, excoriation, and lichenification severity comparable to that of dermatologists [38]. Computational applicative advances in this direction have led to the more recent design of Automatic SCORing Atopic Dermatitis (ASCORAD) [39]. Even more, AI application attempts for classifying AD and subclassifying its severity have also been tested with encouraging results for more recently introduced dermatologic imaging methods, such as 3D Raster-Scanning Optoacoustic Mesoscopy (RSOM), demonstrating a high predictive accuracy in classifying AD for CNNs, but not as accurate for the severity subclassification compared to the RF model [40]. An emerging application thread of AI calls into play its use in assessing how external risk factors are related to the clinical worsening of AD. In this regard, in their observational study, Patella et al., using an ANN for data analysis, found a proportionality between the increased SCORing Atopic Dermatitis (SCORAD) score and increased air pollutant concentration and total pollen count [41]. AI application developments in the past year also involved the evaluation of the outperformance of CNNs, trained with multi-evaluator datasets, in staging AD severity [42]. 

#### 2.1.4. AI in Therapeutic Frontiers in Personalized Medicine

Not only for diagnostic and staging purposes, but also for the assessment of the therapeutic efficacy and therapeutic advancement of AD, even considering the effectiveness of suggested word-embedding-based ML approaches for the new eligibility of existing drugs (drug repositioning), thus paving the way for patient-centered care in the framework of personalized dermatology, AI has revealed its potential through the proposal of a new algorithm that, by integrating actigraphy data with the use of recurrent neural networks, has proven effective in the detection and quantization of nocturnal scratching movements [43,44,45]. Analogous advances have also been attempted in murine models [46]. Far beyond drug repositioning, AI, by employing CNNs, has shown encouraging results in identifying the novel inhibitory molecules (such as caffeoyl malic acid) of AD’s pivotal therapeutic targets [47]. Interestingly, an ML-based analysis has been employed to identify alterations in keratinocyte transcriptomic programs in AD and the impact on them of various drugs including Dupilumab, for which ML analysis has been shown to predict indicators of nonresponse using clinical-demographic data as well as enabling a large-scale investigation regarding the impact of sleep-related adverse reactions to such a biological drug [48,49,50]. Regarding the therapeutic aspect, meanwhile, alongside recently reviewed applications of multiple ML models, an important contribution to precision medicine is offered by the most recent advances regarding the use of new DL-based models capable of generating new drug candidate molecules by employing disease-specific gene expression profiles [51,52]. An aspect entirely in step with the times of self-information and self-management, AI, through platforms such as Chat Generative Pre-Trained Transformer (ChatGPT) and specific mobile health apps, has also begun to play a key role in offering patients access to clinically accurate and inclusive information about this condition, however, not without psychopathological implications, especially in parents of children with AD [53,54,55,56].

- AI, by revealing specific epigenetic dysregulations, has begun to contribute to a better etiopathogenetic understanding of AD [5,6,7,8,9,10].
- AI demonstrated a good performance in predicting disease using both epigenetic data and exposure to environmental factors [15,16,17,18,19].
- The most widely investigated AI application field in AD concerns diagnosis through image recognition and the differential diagnosis with other dermatoses of similar clinical presentation [20,21,22,23,24,25,26,27,28].
- ML models have been extensively used with a good predictive performance of disease severity, even on a daily baseline [37,38,39,40,41].
- ML and DL models have been used with promising results in the therapeutic setting for various purposes, including drug repurposing, the eligibility of new drugs, and the prediction of the therapeutic response to biologic drugs [48,49,50].

### 2.2. Psoriasis

#### 2.2.1. Image Analysis

Psoriasis is a chronic autoimmune skin disorder characterized by the abnormal proliferation of skin cells, considered a T-cell-mediated inflammatory disease. The immune system mistakenly activates T cells which, in turn, stimulate the skin cells to undergo rapid proliferation. This results in the formation of thickened, red patches of skin covered with silvery scales. AI, and more specifically ML, can play a crucial role in the assessment and diagnosis of skin diseases by automatically interpreting skin images. This also involves the capacity to recognize a psoriasis lesion in an image, distinguish it from other skin conditions, outline the contours of the lesion, and assess the severity and extent of psoriasis based on the image. On this topic, Shrivastava et al. have conducted studies focusing on classifying skin images from psoriasis patients as either healthy or diseased, achieving an accuracy of approximately 99% after extracting feature information like texture, color, and redness from the images [57,58,59]. Other research groups have concentrated on differentiating psoriasis from images depicting various common skin disorders, including those often mistaken for psoriasis such as AD or seborrheic dermatitis [11,60,61,62]. For instance, Zhao et al. employed CNNs to classify 8021 images of nine common disorders from a Chinese hospital’s patients, achieving a superior performance compared to 25 Chinese dermatologists when tested on 100 new images [60]. In the same context, other authors have explored the application of dermatoscopic images, in addition to skin images, for AI-based diagnosis, using a DL model. A novel diagnostic method was developed to distinguish between scalp psoriasis and seborrheic dermatitis, which reached a higher accuracy compared to dermatologists trained with dermoscopy [63]. Other research groups have integrated skin computed tomography and confocal laser scanning microscopy with AI algorithms for examining psoriasis. The results indicated a high specificity and sensitivity for features like psoriasis-like hyperplasia and Munro microabscess, providing valuable diagnostic clues, especially in pediatric cases [64]. 

#### 2.2.2. AI-Assisted Severity Scores and Comorbidities

After psoriatic lesion identification, the next step was to evaluate the gravity score of psoriasis based on skin images, to better choose the tailored therapy for the patients. The Psoriasis Area and Severity Index (PASI), Body Surface Area (BSA) and Physician Global Assessment (PGA) systems are scores used worldwide to grade the severity of psoriasis. These grading systems entail the clinical evaluation of lesion erythema, scaliness, and induration. ML techniques have been utilized to automatically assess erythema and scaliness from images and detect changes in scaliness over time in a series of images, achieving a good accuracy for erythema and scaliness evaluation [65,66,67,68]. BSA is another quantitative metric assessed by dermatologists during the evaluation of psoriasis patients, traditionally conducted through a full-body skin examination. ML researchers are actively working on automating the estimation of BSA, achieving an accuracy of over 90% in the images analyzed, with automated area estimates differing from physicians’ estimates by an average of 8.1% [69]. Furthermore, total body imaging systems are being developed to generate more comprehensive images for automatic PASI and BSA measurements [70]. Collectively, the information derived from ML-automated severity and area grading can be utilized for the automatic risk stratification of psoriasis lesions. Some studies suggested that psoriasis can be subject to sudden flares, regardless of the severity of the disease and patient’s characteristics, in the presence of triggers, such as infections or vaccinations. Therefore, the development of AI methods to predict such flares can be of significant importance [71]. AI programs have also been utilized in early detection studies of comorbidities in psoriasis, including psoriatic arthritis (PsA), cardiovascular disease, and diabetes [72,73]. Other studies utilized ML models based on blood immune profiling and serum proteomics to distinguish between PsA and psoriatic patients. These models contributed to the development of a predictive model for minimal disease activity, with global pain, disease impact (PsAID), patient global assessment, and physical function being significant variables [74]. Beyond PsA, patient records were examined to identify top predictors of noncalcified coronary plaque burden in psoriasis, including obesity, dyslipidemia, and inflammation factors [75]. 

#### 2.2.3. AI-Based Therapies and Efficacy Prediction

The subsequent step of AI was to tailor psoriasis treatment based on individual clinical phenotypes, which is a crucial but unmet need with the potential aim to significantly enhance patients’ quality of life and functional capabilities [76]. Predicting the effectiveness of drugs can play a key role in developing and implementing personalized treatment schedules. On this topic, Tomalin et al. utilized statistical and ML techniques to forecast drug efficacy in psoriasis, creating a classifier that predicts whether a patient will respond favorably to tofacitinib or etanercept treatment after 12 weeks [77]. This prediction was based on blood samples detecting 92 inflammation-associated proteins and 65 proteins associated with cardiovascular disease. Similarly, an Italian study developed an ANN model to assess the so-called “fast responder” profiles among psoriatic patients treated with secukinumab, an inhibitor of interleukin (IL)-17A, achieving an overall accuracy of 91.88% [78]. In addition to blood samples, other studies explored various predictors to forecast treatment efficiency, revealing factors like secukinumab dosage, prior anti-tumor necrosis factor (TNF) treatment, methotrexate usage, baseline enthesitis, PsA disease duration, and PASI score [79,80]. Conversely, other researchers also investigated the use of ML to predict the “long-term responses” to biologics, finding that generalized linear models (GLMs) outperformed other models in terms of accuracy and computational efficiency [20]. Finally, in the perspective of enhancing psoriasis treatment outcome, the development of new drugs is crucial [81]. Being a multifactorial genetic disorder with about 70% of its susceptibility attributed to genetic factors, psoriasis makes it mandatory to understand its genetic basis to unravel the disease’s biology, to identify clinical biomarkers, and, above all, to discover new drug targets, with the final aim being to advance toward personalized medicine [82]. An alternative, a cost-effective strategy is drug repurposing, which involves identifying drugs with potential applications beyond their original uses [83]. On this topic, Patrick et al. devised a system to pinpoint drugs suitable for repurposing in psoriasis treatment [43]. Using word embedding to summarize information from over 20 million articles on drugs and applying ML to model drug–disease relationships, the approach successfully identified budesonide and hydroxychloroquine as potential candidates. However, the data at our disposal are still limited to propose, from a clinical standpoint, the use of these drugs in patients who, nonetheless, may benefit from medications already on the market and whose efficacy has already been proven. In conclusion, ML has considerable potential to improve various aspects of psoriasis management, including diagnosis and treatment. In diagnostics, ML can automate tasks like identifying psoriasis-affected areas in photos, differentiating psoriasis from other skin disorders, and quantifying disease severity, thus helping dermatologists’ tasks, especially in high-volume practices. In therapy and management, ML is crucial for preventing complications. For example, ML predictions of characteristics associated with a higher risk of cardiovascular complications can guide the targeting of preventive cardiology services. Moreover, ML enhances psoriasis treatment by automating lesion evaluation. A high PGA computed by ML can prompt dermatologists to consider more intensive systemic treatment or phototherapy instead of topicals. ML also predicts long-term treatment responses, identifies potential drug interactions, and anticipates new therapies for psoriasis.

- ML is a new tool for the diagnosis of psoriasis, with a high accuracy in classifying skin images [57,58,59].
- ML techniques are actively involved in automating the assessment of psoriasis severity using established metrics like PASI and BSA [65,66,67,68,69,70].
- ML is being tested for the early detection of comorbidities associated with psoriasis, through blood immune profiling and serum proteomics [72,73,74].
- ML plays a pivotal role in tailoring psoriasis treatment based on individual clinical phenotypes. This personalized approach aims to enhance treatment outcomes and improve patients’ quality of life [77,78,79,80].

### 2.3. Alopecia Areata

Alopecia areata (AA) is an autoimmune disorder characterized by the sudden onset of non-scarring hair loss in localized or widespread areas on the scalp, face, or body. In individuals with AA, the immune system mistakenly attacks the hair follicles, leading to hair loss. This condition can manifest as small, round patches of hair loss (AA), complete loss of scalp hair, alopecia totalis (AT), or total loss of body hair, alopecia universalis (AU) [84]. The precise cause of AA is not fully understood, but it is believed to involve a combination of genetic, environmental, and immunological factors. The condition is diagnosed clinically and dermoscopically, even though AI is gaining an increasing role. In 2021, a framework was developed to distinguish between healthy hair and AA through image classification, using healthy hair images and AA images, and applying image preprocessing and feature extraction. Using SVM and k-nearest Neighbor (KNN) classification techniques, a reported accuracy of 91.4% for SVM and 88.9% for KNN was achieved. These findings highlight the potential for improved prediction capabilities in the field of dermatology [85]. AI has been also tried in the severity assessment of AA through a DL framework, specifically targeting the Severity of Alopecia Tool (SALT) score. Dermatologists’ naked-eye assessments were compared to the model, called AloNet, with a strong agreement between the dermatologists’ evaluation and the model. Notably, the model exhibited a superior performance in cases of patchy or multifocal alopecia and effectively rejected irrelevant structures in predicting regions of interest [86]. These, although they are the initial data, confirm the ability of AI in diagnostic support for dermatologists in the trichology field. AI has also been used for prognostic purposes. On this topic, a study focused on identifying biomarkers associated with the progression of AA to the subtypes of AT or AU, through bioinformatics analyses on human scalp skin biopsy specimens and the subsequent identification of key genes in AA tissues, particularly in AT and AU subtypes. The findings suggest the importance of these models in guiding the clinical management for different AA patients [84]. Moving to therapy, the challenge of determining the most effective therapeutic approach for each patient is particularly pronounced across various diseases. In the realm of autoimmune diseases like AA, different Janus kinase (JAK)/STAT inhibitors have shown efficacy in clinical trials, each exhibiting distinct response rates. The AI found its application in the study of the variability in patient response. A computational model predicted the likelihood of response for specific patient–drug pairs by integrating the inferred mechanism of action data and gene regulatory networks. This integration incorporates insights from an independent patient cohort, aligning with baseline patient data before the initiation of treatment [87]. In conclusion, AI in AA is finding increasing application both in diagnostics, through the analysis of specific patterns for accurate diagnosis, and in the selection of the most effective treatment for the patient, thus creating personalized therapy. Furthermore, it plays a role in assessing prognosis.

- AI plays a role in diagnosis with image classification [85].
- AI is playing a pivotal role in tailoring treatment strategies for AA patients [87].

### 2.4. Vitiligo

Vitiligo is a chronic skin disorder characterized by the loss of melanocytes, resulting in white patches on the skin. The exact cause is unclear, but factors such as genetics, autoimmunity, and environmental triggers may play a role. Melanocyte destruction, potentially due to autoimmune reactions, leads to depigmentation [88]. In vitiligo, AI has been applied both for diagnosis, prognosis, and therapeutical choice. To assess the severity of vitiligo, AI systems have been developed by comparing them with traditional assessment methods used by dermatologists. The AI models demonstrated an impressive accuracy in assessing the severity of vitiligo, and the comparative analysis with scores assigned by dermatologists showed a good agreement between the scores assigned by human evaluators and the AI model. This evidence suggests that AI models have potential as an objective tool for vitiligo assessment, offering a valid alternative or complement to human assessment in clinical practice and research [89,90]. The use of AI in vitiligo has found application also in phytotherapy, the branch of medicine that uses plants in their entirety or their components for medical purposes to treat or prevent several diseases [91]. Wang et al. proposed a systematic framework for discovering potential therapeutic targets and understanding the mechanism of kaempferide, a major ingredient from Vernonia anthelmintica, for vitiligo. Transcriptome and protein–protein interactome data were collected, and a combination of RF and greedy articulation points removal (GAPR) methods was employed. The RF model demonstrated a good performance, leading to the prioritization of 722 important transcriptomic features, while the network analysis identified 44 articulation proteins in the vitiligo network as potential therapeutic targets using the GAPR method. Integrating these results with the proteomic profiling of kaempferide revealed a multi-target strategy for vitiligo, including the suppression of the p38 mitogen-activated protein kinase (MAPK) signaling pathway and modulation of cellular redox homeostasis. This approach provides a novel perspective for discovering drug candidates and potential therapeutic strategies for vitiligo, demonstrating the utility of the proposed framework in complex disease research [92].

- AI models, when compared with traditional assessment methods used by dermatologists, demonstrated an impressive accuracy [89,90].
- A multi-target strategy for vitiligo assessment is an object of study [92]

### 2.5. Hidradenitis Suppurativa

Hidradenitis suppurativa (HS) is a chronic skin condition characterized by the formation of painful nodules, abscesses, and tunnels beneath the skin, primarily in areas where skin rubs together, such as the armpits, groin, buttocks, and under the breasts [93]. This condition involves the inflammation of hair follicles and apocrine sweat glands. The exact cause of hidradenitis suppurativa is not fully understood, but factors like genetics, inflammation, and hormonal influences may contribute to its development. AI is finding a plethora of applications in HS, including diagnostic support, monitoring and management, patient education and support, and, finally, predictive analysis, helping healthcare providers anticipate disease progression and tailor treatment plans accordingly. The major application of AI is the aim to overcome the inter-variability in the assessment of the patient’s disease stage. On this topic, to overcome the International Hidradenitis Suppurativa Severity Score System (IHS4), which is time-consuming and subject to variability, the Automatic International Hidradenitis Suppurativa Severity Score System (AIHS4) is introduced, using a DL model, Legit. Health-IHS4net, for lesion detection. The results indicate that the AIHS4 can assess the severity of HS in a manner comparable to expert clinicians, suggesting its potential implementation in CAD systems. This evidence highlights the utility of AI in evidence-based dermatology, offering a potential tool to empower dermatologists in daily practice and clinical trials [94].

- AIHS4 can evaluate the severity of HS like expert clinicians, indicating its potential integration into CAD systems [94].

### 2.6. Acne

Traditionally, patients seeking an acne diagnosis must physically visit a dermatologist, where the expert assesses affected areas either visually or with a dermatoscope. The diagnostic process heavily relies on the dermatologist’s expertise and experience [95]. However, the scarcity of dermatologists in various regions forces many patients to endure long journeys or extended wait times. Recent strides in smartphone technology, embraced by approximately 3.2 billion people globally, have paved the way for innovative healthcare solutions. One notable example is teledermatology, which allows patients to receive remote consultations via smartphones, eliminating the need for in-person visits and saving valuable time [96]. Especially after the COVID-19 pandemic, teledermatology is proving beneficial for individuals living in rural or distant areas, enhancing access to dermatological care [97,98]. Simultaneously, the ongoing research in the development and integration of highly precise, automatic skin image analysis algorithms aims to assist doctors in expediting diagnoses and furnishing valuable information to patients [99]. These algorithms have the potential to streamline the diagnostic process, enhancing both efficiency and accuracy. The intersection of these advancements in dermatology and teledermatology signifies a dynamic field of exploration. The goal is to harness the benefits of smartphone-based solutions, making dermatological care more accessible, particularly for those in underserved or remote communities [96,99]. Numerous skin image analysis algorithms, specifically designed for acne analysis, have emerged as part of this ongoing research and technological evolution. On this topic, AI-powered acne-grading systems that incorporate lesion identification and assess its performance compared to physician image-based scoring have been tested, with the AI severity-grading system showing a good agreement with the true label. Moreover, the integration of lesion identification into severity assessment improved agreement, suggesting potential clinical decision support effectiveness [100]. To address the challenges associated with existing acne-grading methods, such as variability among raters and time-consuming lesion counting, new automatic acne lesion counting programs have been developed and optimized to characterize the subtypes of acne. The AI-based programs demonstrated favorable results in the sensitivity and positive predictive value for papules, nodules, pustules, and whitehead comedones when compared to manual counting by an expert dermatologist. The findings suggest the usefulness of the automatic lesion-counting program in efficiently assessing acne lesions [101]. The performance of AI was also observed in the evaluation of acne severity. The AI, trained on images, demonstrated a high correlation with the assessments made by clinicians following the Investigator’s Global Assessment (IGA) scale. This marks the first case where AI has directly classified acne patients according to the IGA ordinal scale with a high accuracy, eliminating the need for human intervention [102,103]. The results demonstrated the potential of AI and large datasets for the automated analysis and classification of clinical images, offering a standardized approach to assessing acne severity. Since the complexity of skin lesions often limits the effectiveness of conventional image-processing methods, numerous algorithms for analyzing skin images have been developed [104,105]. The introduction of DL techniques, particularly CNNs, has significantly advanced the field of computer vision in skin image analysis. Recent studies have explored the application of DL to improve upon the limitations of traditional image-processing approaches in acne analysis, through the development of models capable of successfully distinguishing the various classes of acne lesions (blackheads, pimples, papules, pustules, nodules, cysts, and normal skin) with a high accuracy [106]. Among these models, the AcneNet model, using a deep residual neural network, achieved an impressive overall accuracy of over 94% [107]. A subsequent step was taken by Seite et al., who presented a DL-based AI algorithm for facial acne analysis using smartphone images [108]. This method could evaluate the severity of facial acne based on the Global Evaluation Acne (GEA) scale and identify various types of acne lesions. However, the accuracy in classifying acne severity provided by the method was 68%. In 2021, Yin Yang et al. developed another acne rating algorithm using DL to classify the severity of acne on the face according to Chinese guidelines, which demonstrated a strong correlation between the model and dermatologists [109]. Finally, in 2022, Liu et al. introduced a new overall pruning framework to accurately detect and classify acne using DL models. The proposed method involved training multiple base models and eliminating redundant models based on performance and diversity. The framework achieved a high prediction accuracy of 85.82% on the acne dataset, surpassing the results achieved by existing studies. The approach was also tested on a skin cancer dataset and demonstrated a superior performance compared to state-of-the-art methods [110]. In summary, the confluence of advancements in dermatology, teledermatology, and DL techniques has paved the way for a more accessible and efficient acne diagnosis and severity assessment, particularly by leveraging the widespread use of smartphones globally. Ongoing research continues to refine and enhance these technologies for the benefit of patients and healthcare providers alike. 

Teledermatology, facilitated by the widespread adoption of smartphones, has emerged as a new solution for acne diagnosis [96].

- The integration of AI-powered acne-grading systems marks a significant advancement in automating the diagnostic process [100,101].
- The application of DL techniques, particularly CNNs, has revolutionized the acne severity assessment. Models like AcneNet, utilizing deep residual neural networks, have achieved a remarkable overall accuracy of over 94% [107].

### 2.7. Rosacea

In recent years, it has become clear how the application of AI and, specifically, of deep CNNs through image recognition, further refined with data augmentation techniques, plays no small contribution in the diagnostic facilitation of rosacea and other dermatopathies with a high incidence and similar clinical presentation with the increasingly cutting-edge proposition of frameworks representative of authentic dermatologic clinical practice within defined geographic boundaries [81]. In the wake of such trends, Binol et al. proposed Ros-NET, a new framework for the automatic identification of rosacea lesions based on deep-learning and transfer-learning systems by using deep CNNs pre-trained with query patches obtained from photographic facial images in addition to a new anthropometric model. The ultimate result was a significant decrease in the rate of false positives revealed [111]. Further confirmation about the deciphering capabilities from clinical images of deep CNNs comes from Zhao et al., in whose study the use of ResNet-50 proved effective and accurate in identifying rosacea by discerning it from other facial dermatoses of similar clinical presentation, as well as classifying it into the three major subtypes. In addtion, the identification performance of their model was comparable to that of an experienced dermatologist [112].

- The state-of-the-art AI clinical applications for rosacea concern only its diagnostic facilitation by the automatic identification and classification of subtypes from facial images [111,112].

### 2.8. Lichen

**Figure 1:**
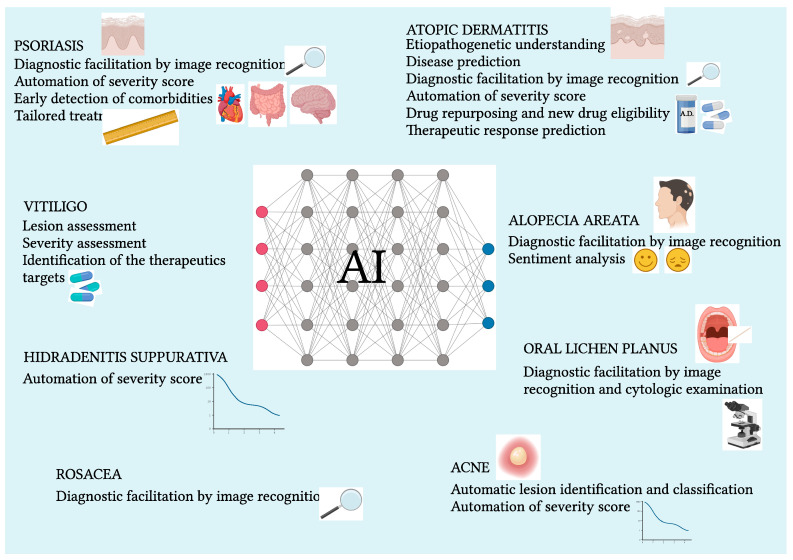
The main findings about the role of AI in chronic inflammatory skin diseases are described. Created with BioRender.com.

The use of AI for efficient decision-making support, especially in real clinical settings and for predictive purposes in the diagnosis of oral lichen planus (OLP) and lichen planus-like keratosis, has been pioneered in the past decade through the use of ML algorithms [81,113,114,115]. The potentially useful role of interleukin 12 receptor beta 2/tumor necrosis factor receptor superfamily member 8 (IL12RB2/TNFRSF8) ratio as a biomarker in the differential diagnosis between OLP and other chronic nonspecific mucositis has emerged [116]. The predictive model just discussed helped to pave the way for the further use of ML techniques in the dichotomous classification of patients with OLP, as part of the evaluation regarding the diagnostic potential of their salivary cytologic profile [117]. Likewise, the creation of ANNs capable of detecting and quantifying the presence of monocytes and granulocytes in the inflammatory infiltrates typical of OLP has made it possible to define a distinctive cut-off threshold between OLP and other lichenoid lesions, thus representing a valuable aid for anatomopathologic diagnosis [118]. Moreover, in more recent times, the predictive accuracy of lichen planus and other skin diseases has been increasing, starting from a more efficient use of datasets, made possible by the development of new ML algorithms capable of combining various and individual data-mining techniques, under the so-called “multi-model ensemble method” [61]. The latest AI employment frontier in the OLP diagnostic facilitation calls into play deep CNNs capable of distinguishing between OLP and non-OLP lesions with an 82% to 88% accuracy [119]. In the past year alone, the literature has been teeming with work related to the application of AI, both in evaluating the performance of new DL-based models in OLP lesion identification, as well as making initial use of the assisting function of ChatGPT for conceptual assimilation and synthesis and for drafting, and then the qualitative improvement of the manuscript related to a retrospective study on lichen sclerosus et atrophicus (LSEA) [120,121]. Figure 1 shows the role of AI in chronic inflammatory skin diseases.

- The current AI applications for OLP exclusively concern its diagnostic (including cytologic) facilitation and differential diagnosis from other chronic nonspecific mucositis and lichen planus-like keratoses, especially through the identification of new potential biomarkers [61,117,118,119].

General

- Except for immunobullous diseases, clinical applications of AI are being widely investigated for all other major chronic dermatoses.
- Application areas involve the improved understanding of etiopathogenesis, prediction of disease onset, automation of diagnosis and differential diagnostic by image recognition, identification of new biomarkers for diagnostic and prognostic purposes, characterization of pheno-endotypes and subtypes of disease, early identification of comorbidities, automation of disease severity staging, drug repositioning, identification of new drug candidates, and prediction of therapeutic response.
- The dermatoses of major interest so far are atopic dermatitis, psoriasis, and acne.
- Future and challenging AI application goals towards a “precision intelligence” concern the ever-increasing mastery of epigenetic datasets for the unequaled clinical-therapeutic cognitive evolution of discussed dermatoses.

## 3. Conclusions

AI is increasingly dominating, year after year, not only our daily lives but, also, and above all, the international scientific scene. Indeed, a multitude of increasingly improved clinical application fields are emerging: the prediction of the onset, better definition of the etiopathogenesis, diagnosis, prognosis, and management of a wide variety of diseases. In our proposed narrative review, the intent has been to examine the most cutting-edge application evidence of the various AI subsets in the extensive domain of chronic inflammatory and autoimmune dermatoses, in this meaning ever more objectively and meticulously assessed and understood, especially by reason of their huge impact, as well as the socio-economic aspect, on the quality of life of patients and caregivers. As if that were not enough, the new frontier AI promises to reach and master ever more thoroughly the massive body of transcriptome and microbiome data; these latter are parallel but have as intertwined information universes as ever, with the ambitious goal of identifying new biomarkers of disease, as well as predicting its occurrence ever more accurately. Early exploratory evidence in this matter is limited to the analysis of data from the intestinal microbiome. But, while remaining on topic with the subject of our discussion, it would be interesting, for future research purposes, to be able to assess AI potential applications concerning the investigation of skin microbiome data. Last but not least, to the increase technological implementation and decisional transparency, a not negligible aspect needs to be better defined: the explainability of AI algorithms. These, in fact, while returning outputs coherent with inputs, due to their data processing complexity, do not allow the human mind to understand why certain decisions are made. Such “cognitive opacity” has, in fact, led to AI algorithms that resembled veritable “black boxes”. In this sense, the opening of the black box, in other words, the full understanding of how the given input data are processed by the algorithm and transformed into a result (output), would be indispensable for the preservation of the scientific method as an essential pillar of truthful knowledge about reality. The challenge of modern explainable AI, even and especially in the clinical setting, is to achieve transparent results while not sacrificing the statistical significance that would be achieved by keeping the black box closed. Besides the just-discussed technological limitation, for the future purposes of AI development in this dermatological setting, also noteworthy are the major limitations of different backgrounds: clinical, cultural, and socio-economic. For the first one mentioned, it will certainly be necessary to determine which dermatoses need a higher degree of accuracy than others, to what extent, such that a rigorous statistical analysis is still preferable to an algorithm-based approach. Cultural limitation, on the other hand, calls into question the reluctance of the current class of dermatologists to have their clinical knowledge, fine-tuned over years of visual experience, challenged and sometimes outclassed, or to lose autonomy in specific areas of clinical decision-making. With this in mind, one wonders how profitable the emerging human–machine partnership can be. Last but not least, the socio-health limitation intuitable to date would presume more elucidation of possible disparities in terms of healthcare access that AI might create between underdeveloped and industrialized countries, especially in an epidemiologically well-represented field such as chronic inflammatory skin conditions.
